# Predictive biomarkers for disease sensitivity in lymphoma – the holy grail for HDAC inhibitors?

**DOI:** 10.18632/oncotarget.26460

**Published:** 2018-12-18

**Authors:** Toby A. Eyre

**Affiliations:** Toby A. Eyre: Early Phase Trials Unit, University of Oxford, Oxford, UK; Department of Clinical Haematology, Oxford Cancer Centre, Churchill Hospital, Oxford, UK

**Keywords:** HR23B, HDAC inhibitor, T cell lymphoma, Hodgkin lymphoma, CXD101

Histone deacetylase inhibitors (HDACi) have been studied across a range of malignancies in recent years with variable success. The key putative mechanisms of action of HDAC inhibitors are debated but include the hyperacetylation of nucleosomal histones, which results in tightened chromatin coiling with resultant silencing of gene expression. This process is likely critical the processes of cell survival regulation, proliferation, differentiation, and apoptosis [1]. Additional documented cellular mechanisms include the induction of pro-apoptotic genes and their protein expression and interference with tyrosine kinases and steroid receptors [2].

The success of HDAC inhibitor monotherapy has largely been limited to patients with relapsed, refractory (R/R) Hodgkin lymphoma and certain subtypes of non-Hodgkin lymphoma, in particular, peripheral T cell lymphoma (PTCL) and cutaneous T cell lymphoma (CTCL). Examples of these studies include two phase II studies [3, 4] which showed an overall response rate (ORR) of 25-34% with romidepsin monotherapy in R/R PTCL and CTCL and in a separate phase II trial of 129 R/R PTCL patients [5] which demonstrated an ORR of 25.8% with belinostat, a class-I-II-IV HDACi. Similar results also are documented with chidamide [6] in relapsed PTCL. Panobinostat has demonstrated an ORR of 27% (CR 4%) in patients with Hodgkin lymphoma relapsing in the post autologous stem cell transplantation (ASCT) setting [7]. In contrast, outcomes in patients with solid non-haematological malignancies have be poor and no agent is licensed outside the lymphoma indications listed.

Taken together, responses occur in approximately 25-33% in the disease indications discussed. Perhaps mostly striking is that in the patients that do respond, their duration of response is often particularly long lasting within the clinical context. HDAC inhibitor toxicities can be problematic, with HDACi-related discontinuation rates at approximately 10-15% in clinical trials with the main side effects including cytopenias, nausea, diarrhoea and fatigue. Given the potential toxicity associated with HDAC inhibitors, the lack of response in 66-75% of patients and the remarkable durability of some responses documented, one could argue that this class of anti-cancer therapy represents the perfect setting for the investigation of a predictive biomarker.

A genome-wide loss-of-function screen has previously identified the protein HR23B, which functions to shuttle ubiquitinated cargo proteins to the proteasome for degradation as a sensitivity determinant for HDACi-induced cell apoptosis [8]. Proteasome activity is thought to be deregulated by HDAC inhibition *via* a HR23B-dependent mechanism. Manipulation of HR23B levels within CTCL cells *in vitro* has been shown to alter HDACi sensitivity [9]. Furthermore, high HR23B expression in patients with R/R CTCL treated in a trial of romidepsin monotherapy has correlated clearly with responses [4]. Taken together, these findings provided a rationale for testing the hypothesis that HR23B may provide a predictive biomarker for durable responses to HDAC inhibition.

CXD101 is a novel class I-selective HDACi (HDAC1 (63nM inhibitory concentration; IC-50), HDAC2 (570nM IC-50), HDAC3 (550nM IC-50)). CXD101 has no activity against HDAC class II (Celleron data). It was hypothesised that the toxicity profile of CXD101 *may* be improved compared to other licensed HDAC inhibitors by minimising class II target effects, such as the potential for cardiac toxicity, whilst retaining anti-tumour activity as shown with other agents with class I activity.

The initial results of the dose escalation study of CXD101 in patients with advanced relapsed, refractory cancer were recently published by Eyre and colleagues in the journal Cancer [10]. The maximum tolerated dose and recommended phase II dose of 20 mg for 5 days in a 21-day cycle was established following a 3+3 dose escalation design. 30 patients were dosed during the escalation phase and 6 patients in a small expansion phase.

Early results from an important sub analysis of 17 heavily pre-treated R/R lymphoma patients treated (at 16 mg or over) demonstrated an ORR of 23.5% (4/17) with 6 other patients benefiting from meaningful stable disease with reduction in tumour volume. The 4 responding patients (3 partial response, 1 complete response) had demonstrable durability of response, with a duration of response in each patient of 203 days, 161 days, 173 days, and 441 days respectively. CXD101 displayed a favourable safely profile. Key grade 3 to 4 adverse events (according to Common Terminology Criteria for Adverse Events criteria (version 4.03)) included thrombocytopenia (11%), neutropenia (17%), and neutropenic fever (2%) across the total of 133 CXD101 cycles given.

HR23B expression by immunohistochemistry was not clearly associated with overall response or progression-free survival in this very preliminary assessment, which was limited by dose and tumour subtype variability. Feasibility for prospective testing of HR23B by immunohistochemistry was demonstrated within the trial, and a further analysis is planned within a larger, more homogenous cohort of R/R peripheral T cell lymphoma patients being currently investigated in the ROMICAR trial (romidepsin-carfilzomib in R/R PTCL (NCT03141203)). We await the assessment of HR23B in this and other analyses with great interest.

This phase I trial is the first to integrate a prospective, pre-planned biomarker analysis within a clinical trial design investigating HDAC inhibition in advanced malignancies. Given the duration of responses seen with HDAC inhibition, it represents a prime example of where a robust and reliable predictive biomarker would be of great benefit to help clinicians target treatment in pre-selected patients. HR23B has potential to provide this as a simple, easily applicable, immunohistochemistry test performed on recent tumour biopsies, however this test requires further robust analysis in larger cohorts before a definitive conclusion of its utility can be formally reached.
