# Correction: Predictive biomarkers for disease sensitivity in lymphoma – the holy grail for HDAC inhibitors?

**DOI:** 10.18632/oncotarget.26767

**Published:** 2019-02-26

**Authors:** Toby A. Eyre

**Affiliations:** Early Phase Trials Unit, University of Oxford, Oxford, UK; Department of Clinical Haematology, Oxford Cancer Centre, Churchill Hospital, Oxford, UK

**This article has been corrected:** The correct 10 th reference is given below:

10. Eyre TA, et al. Cancer. 2019; 125:99-108. https:doi.org/10.1002/cncr.31791.

Oncotarget. 2018; 9:37280-37281. https://doi.org/10.18632/oncotarget.26460

